# Biomechanical assessment of the effects of decompressive surgery in non-chondrodystrophic and chondrodystrophic canine multisegmented lumbar spines

**DOI:** 10.1007/s00586-012-2285-0

**Published:** 2012-04-11

**Authors:** Lucas A. Smolders, Idsart Kingma, Niklas Bergknut, Albert J. van der Veen, Wouter J. A. Dhert, Herman A. W. Hazewinkel, Jaap H. van Dieën, Björn P. Meij

**Affiliations:** 1 Department of Clinical Sciences of Companion Animals, Faculty of Veterinary Medicine, Utrecht University, Yalelaan 108, PO Box 80.154, 3508 TD Utrecht, The Netherlands; 2 Research institute MOVE, Faculty of Human Movement Sciences, VU University, Van der Boechorststraat 9, 1081 BT Amsterdam, The Netherlands; 3 Division of Small Animals, Department of Clinical Sciences, Faculty of Veterinary Medicine and Animal Sciences, Swedish University of Agricultural Sciences, Ulls väg 12, Box 7040, 750 07 Uppsala, Sweden; 4 Department of Orthopedic Surgery, VU University Medical Center, Research Institute MOVE, De Boelelaan 1117, 1007 MB Amsterdam, The Netherlands; 5 Division of Surgical Specialties, Department of Orthopaedics, University Medical Center Utrecht, Heidelberglaan 100, 3584 CX Utrecht, The Netherlands

**Keywords:** Biomechanics, Lumbar spine, Intervertebral disc degeneration, Canine model, Nucleotomy

## Abstract

**Purpose:**

Dogs are often used as an animal model in spinal research, but consideration should be given to the breed used as chondrodystrophic (CD) dog breeds always develop IVD degeneration at an early age, whereas non-chondrodystrophic (NCD) dog breeds may develop IVD degeneration, but only later in life. The aim of this study was to provide a mechanical characterization of the NCD [non-degenerated intervertebral discs (IVDs), rich in notochordal cells] and CD (degenerated IVDs, rich in chondrocyte-like cells) canine spine before and after decompressive surgery (nucleotomy).

**Methods:**

The biomechanical properties of multisegmented lumbar spine specimens (T13–L5 and L5–Cd1) from 2-year-old NCD dogs (healthy) and CD dogs (early degeneration) were investigated in flexion/extension (FE), lateral bending (LB), and axial rotation (AR), in the native state and after nucleotomy of L2–L3 or dorsal laminectomy and nucleotomy of L7–S1. The range of motion (ROM), neutral zone (NZ), and NZ stiffness (NZS) of L1–L2, L2–L3, L6–L7, and L7–S1 were calculated.

**Results:**

In native spines in both dog groups, the greatest mobility in FE was found at L7–S1, and the greatest mobility in LB at L2–L3. Surgery significantly increased the ROM and NZ, and significantly decreased the NZS in FE, LB, and AR in both breed groups. However, surgery at L2–L3 resulted in a significantly larger increase in NZ and decrease in NZS in the CD spines compared with the NCD spines, whereas surgery at L7–S1 induced a significantly larger increase in ROM and decrease in NZS in the NCD spines compared with the CD spines.

**Conclusions:**

Spinal biomechanics significantly differ between NCD and CD dogs and researchers should consider this aspect when using the dog as a model for spinal research.

## Introduction

Low back pain is a common ailment with a considerable socioeconomic impact [1, 2]. A major cause of low back pain is degeneration of the intervertebral disc (IVD) [3, 4]. IVD degeneration is also an important cause of back problems in dogs, and the underlying processes show many similarities in dogs and humans [5]. For these reasons, dogs are often used to study spinal biomechanics and strategies to reverse or ameliorate IVD degeneration [6, 7]. However, it should be borne in mind that various dog breeds are not uniformly susceptible to developing IVD degeneration. With regard to IVD degeneration, dogs can be divided into chondrodystrophic (CD) (e.g., Beagle) and non-chondrodystrophic (NCD) dog breeds (e.g., mongrel dog). The CD dogs are characterized by abnormal growth of the long bones due to a genetic disorder, resulting in short limbs relative to the length of the spine, whereas the NCD dogs exhibit a normal growth of the long bones. CD dogs develop IVD degeneration at about 1 year of age. In contrast, in NCD dogs, signs of IVD degeneration are generally seen later in life, at 6–8 years of age [8]. Moreover, significant differences in the cell population (NCD: rich in notochordal cells; CD: rich in chondrocyte-like cells), matrix composition, and morphology of the nucleus pulposus (NP) can be seen at an early age in the two types of dog [8–10].

The aim of surgery for IVD degeneration-related diseases is to relieve the compression of neural structures and may consist of removal of the NP (nucleotomy) alone or combined with partial removal of the vertebral roof (laminectomy) [11]. Decompressive surgery results in spinal instability in humans [12]; however, the effects of decompressive surgery in either NCD or CD dogs separately have not been investigated. A biomechanical investigation of the NCD and CD multisegmented spine before and after decompressive surgery may provide insight into the effects of IVD degeneration and surgical interventions on spinal biomechanics. This knowledge should be taken into account when using the dog as a model in future spinal research.

## Materials and methods

Eighteen T13–L7–Sacrum–Cd1 spinal specimens were isolated from nine healthy, female mongrel dogs (NCD: mean weight 17.4 kg, range 16.1–20.4 kg) and nine healthy, female Beagle dogs (CD: mean weight 14.3 kg, range 13.0–17.2 kg). All dogs were euthanized at approximately 2 years of age in unrelated experiments approved by the Ethics Committee on Animal Experimentation of Utrecht University. During their life, the dogs had a normal level of activity. Dorsoventral and lateral radiographs were taken to exclude spinal pathology other than IVD degeneration.

### Specimen preparation and testing

Specimens were prepared and tested as described previously [13]. Immediately after euthanasia, the spinal specimens were harvested. The pelvis was removed and each specimen was sawn in the transverse plane halfway through the L5 vertebra, creating nine T13–L5 and nine L5–Cd1 specimens (note that the canine spine has 13 thoracic and 7 lumbar vertebrae) for both the NCD and CD dog group. The specimens were stored at −20 °C and prior to biomechanical testing each specimen was thawed at 4 °C (24 h) and cleaned of all soft tissues, except for the ligamentous tissues and IVDs. The cranial and caudal segment ends were fixed in neutral orientation in two metal cups, using an alloy with a low melting point (cerro-low147, consisting of 48 % bismuth, 25.6 % lead, 12.8 % tin, 9.5 % cadmium, 4 % indium; Cerro Metal Products Co., Bellefonte, PA, USA). During preparation and testing, the specimens were kept moist by regularly spraying them with saline solution (0.9 % NaCl). The fixed spinal specimens were inserted into a four-point bending device [13]. Load was applied with hydraulic materials testing machine (Instron Model 8872, Instron Corporation IST, Toronto, ON, Canada) attached to the four-point bending device. L-shaped flags, each containing three light-emitting diodes (LEDs), were attached to the ventral side of each vertebral body (L1, L2, and L3 in T13–L5 specimens; L6, L7, and S1 in the L5–Cd1 specimens). The movement of each LED was recorded with an optoelectronic 3D movement registration system (resolution: 0.02 mm) with one array of three cameras (Optotrak 3020, Northern Digital Inc, Waterloo, Ontario, Canada). The sampling rate for both the load and the displacement measurements was 50 samples/s. Each specimen was deformed at a constant displacement rate of 1.0°/s and was subjected to a cyclic bending moment (back and forth from −2 to +2 Nm; a bending moment of 2 Nm was determined to result in physiological displacement of the tested canine specimens), applied in the region of T13 to L5 or L5 to Cd1. Specimens were tested in the following sequence: (1) flexion/extension (FE), (2) lateral bending (LB), (3) axial rotation (AR); specimens were subjected to three loading cycles per motion direction.

### Testing steps

**Fig. 1:**
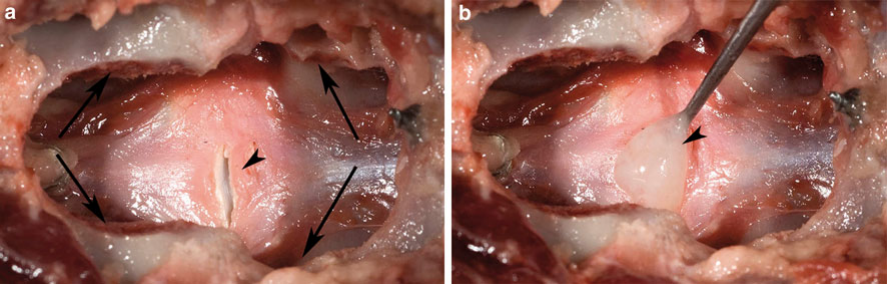
Dorsal view of the L7–S1 segment from a non-chondrodystrophic spine after dorsal laminectomy, showing the laminectomy defect (*arrows*) and the incision in the dorsal annulus fibrosus (*arrowhead*) (**a**). The mucoid nucleus pulposus (*arrowhead*) was removed (nucleotomy) from the nuclear cavity using a ball-tipped probe (**b**)

**Fig. 2:**
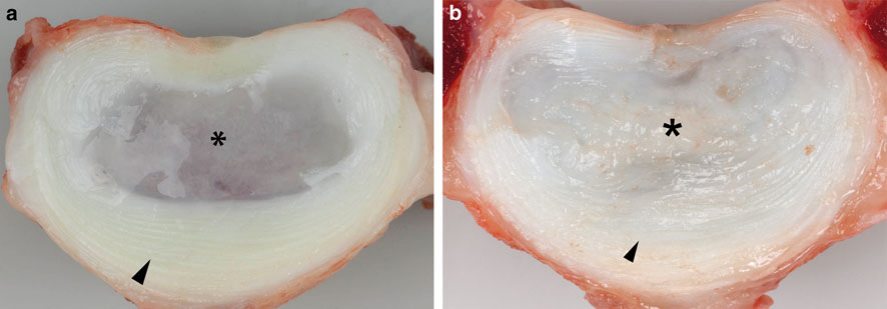
Transverse section of an L2–L3 intervertebral disc obtained from a 2-year-old non-chondrodystrophic dog (**a**) and a 2-year-old chondrodystrophic (**b**) dog, showing the central nucleus pulposus (NP) (*asterisk*) and the outer annulus fibrosus (*arrowhead*)

Each specimen was tested in the native state and after surgery. In the T13–L5 specimens, surgery was performed on the L2–L3 IVD. Via a left lateral approach, a small, transverse stab incision was made into the middle of the IVD with a #11 surgical blade. All NP material was removed (nucleotomy) through the annular incision, using a ball-tipped probe and grasping forceps. The L1–L2 IVD was left intact and served as a control. In the L5–Cd1 specimens, surgery was performed on the lumbosacral (L7–S1) junction via a dorsal approach. The dorsal vertebral arch was removed [dorsal laminectomy; Fig. 1a)] as described previously [13], leaving the articular facets intact. Subsequently, nucleotomy of the L7–S1 IVD was performed as described for the L2–L3 IVD, but through a dorsal annular incision (Fig. 1b). The L6–L7 IVD was left intact and served as a control. The structural composition of all surgically removed NPs was examined macroscopically. The NP taken from the NCD discs was gel-like, similar to the NP seen in Thompson grade I IVDs, whereas the NP from the CD discs had a fibrocartilaginous appearance, similar to the NP seen in Thompson grade II IVDs (Fig. 2) [14].

### Data analysis

Angular displacement of the L1–L2, L2–L3 (T13–L5 specimens) and of the L6–L7, L7–S1 (L5–Cd1 specimens) intervertebral junctions was calculated using the spatial measurements obtained by means of the LED markers, collected with the Optotrak system. Only the data obtained during the third loading cycle of each motion series were analyzed. The following parameters were calculated before and after surgery: Range of motion (ROM), the range of angular displacement between minimum (−2 Nm) and maximum (+2 Nm) applied moments [15]; Neutral zone (NZ), the range over which the specimen moves essentially free of applied loading; NZ was determined by calculating the angular displacement found between −0.1 and +0.1 Nm of applied moment; Neutral zone stiffness (NZS), the quotient of loading and angular displacement in the NZ. NZS was calculated from the upward slope of the load–displacement curve. The ROM, NZ, and NZS were calculated for FE (ROM_FE_, NZ_FE_, NZS_FE_), LB (ROM_LB_, NZ_LB_, NZS_LB_), and AR (ROM_AR_, NZ_AR_, NZS_AR_).

### Statistics

Statistical analyses were performed using R statistical software [16]. The parameters ROM, NZ, NZS were analyzed using a linear mixed model containing both fixed and random effects [17]. The Akaike information criterion (AIC) was used for model selection. The factors incorporated in the fixed part were ‘motion direction’ (FE, LB, AR), ‘condition’ (native spine, operated spine),‘dog type’ (NCD, CD), and the 2- and 3-way interactions between these factors. A random intercept for ‘dog’ (18 spinal segments) was added to take the correlation within each dog into account*.* Normal distribution of the response variables within each model was assessed with PP and QQ plots. In the case of significant interactions between factors, post hoc *T* tests were used to calculate the P values for specific effects of interest. The Benjamini and Hochberg False Discovery Rate procedure was used to correct for multiple testing [18]. P < 0.05 was considered statistically significant.

## Results

The biomechanics of the native spine at different levels was markedly different in both the NCD and CD spines: in both groups, the L7–S1 spinal segment had the highest ROM_FE_, approximately three times higher than that of L1–L2, L2–L3, and L6–L7 (Table 1). The highest ROM_LB_ was found at L2–L3, followed by L1–L2, L6–L7, and L7–S1. The ROM_AR_ was relatively small at all spinal levels and showed little variation between segment levels, but was highest at L7–S1. A similar but more pronounced intersegmental pattern was found for the NZ. The NZS showed a similar, but opposite, pattern compared with that of the ROM and NZ.

**Table 1:** Parameter values of non-chondrodystrophic and chondrodystrophic spines

	ROM		NZ		NZS	
	NCD	CD	NCD	CD	NCD	CD
FE						
L1–L2	10.1 ± 1.72	11.9 ± 1.41	1.41 ± 0.38	1.47 ± 0.42	0.23 ± 0.08	0.15 ± 0.04
L2–L3	9.80 ± 1.72	11.4 ± 1.31	1.59 ± 0.47	1.45 ± 0.52	0.21 ± 0.07	0.16 ± 0.06
L2–L3*	13.0 ± 2.85	14.0 ± 1.78	5.51 ± 1.77	5.93 ± 1.41	0.05 ± 0.03	0.03 ± 0.01
L6–L7	13.7 ± 2.71	14.4 ± 2.64	2.04 ± 0.37	1.89 ± 0.56	0.14 ± 0.02	0.12 ± 0.07
L7–S1	33.6 ± 3.36	38.2 ± 4.07	9.25 ± 2.14	9.93 ± 3.88	0.03 ± 0.01	0.02 ± 0.01
L7–S1*	43.4 ± 3.50	44.8 ± 6.58	21.2 ± 3.83	16.8 ± 3.93	0.01 ± 0.01	0.01 ± 0.01
LB						
L1–L2	15.3 ± 2.18	17.6 ± 1.36	4.07 ± 1.17	3.39 ± 0.57	0.08 ± 0.04	0.06 ± 0.01
L2–L3	21.2 ± 2.77	17.7 ± 2.15	6.59 ± 1.19	3.69 ± 0.89	0.04 ± 0.01	0.06 ± 0.01
L2–L3*	23.5 ± 2.30	22.9 ± 3.60	12.9 ± 2.92	10.3 ± 2.74	0.02 ± 0.01	0.02 ± 0.01
L6–L7	10.6 ± 3.69	9.93 ± 3.38	1.82 ± 0.37	1.20 ± 0.53	0.16 ± 0.04	0.19 ± 0.07
L7–S1	8.10 ± 1.16	10.6 ± 1.66	1.02 ± 0.37	1.36 ± 0.47	0.35 ± 0.15	0.17 ± 0.07
L7–S1*	10.3 ± 1.25	12.2 ± 2.78	3.74 ± 1.03	3.66 ± 1.84	0.07 ± 0.02	0.06 ± 0.03
AR						
L1–L2	0.70 ± 0.14	1.28 ± 0.45	0.07 ± 0.04	0.07 ± 0.07	7.61 ± 3.40	5.10 ± 4.51
L2–L3	0.92 ± 0.23	1.29 ± 0.34	0.08 ± 0.03	0.06 ± 0.05	5.37 ± 1.83	3.62 ± 1.23
L2–L3*	1.32 ± 0.40	2.12 ± 0.49	0.17 ± 0.07	0.24 ± 0.26	2.78 ± 1.45	1.39 ± 0.82
L6–L7	0.74 ± 0.38	1.28 ± 0.52	0.07 ± 0.07	0.07 ± 0.03	8.25 ± 3.70	4.27 ± 3.63
L7–S1	1.05 ± 0.55	1.81 ± 0.60	0.11 ± 0.06	0.10 ± 0.08	4.67 ± 2.00	2.56 ± 1.61
L7–S1*	2.56 ± 0.88	3.00 ± 0.87	0.35 ± 0.18	0.24 ± 0.10	1.35 ± 0.74	1.01 ± 0.46

Mean ± SD for the parameters range of motion (ROM; degrees), neutral zone (NZ; degrees), and neutral zone stiffness (NZS; Nm/degree) for flexion/extension (FE), lateral bending (LB), and axial rotation (AR) for L1–L2, L2–L3, L6–L7, and L7–S1 of native non-chondrodystrophic (NCD) and chondrodystrophic (CD) spines. L2–L3* (nucleotomy) and L7–S1* (dorsal laminectomy and nucleotomy) depict the parameter values after spinal surgery

**Fig. 3:**
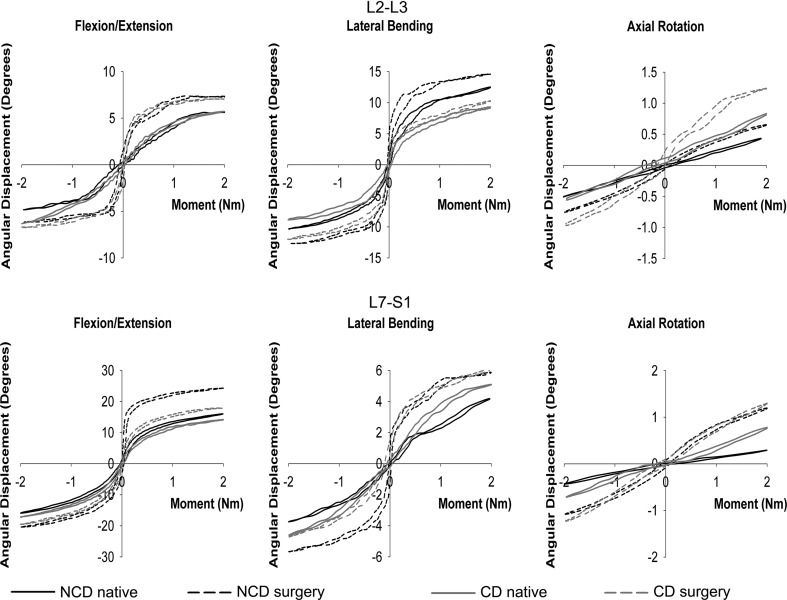
Representative load–displacement curves for non-chondrodystrophic (NCD) and chondrodystrophic (CD) spines in flexion/extension, lateral bending, and axial rotation for the L2–L3 and L7–S1 spinal segment in the native state and after nucleotomy (L2–L3) or dorsal laminectomy and nucleotomy (L7–S1). Only the third loading cycles are displayed

**Fig. 4:**
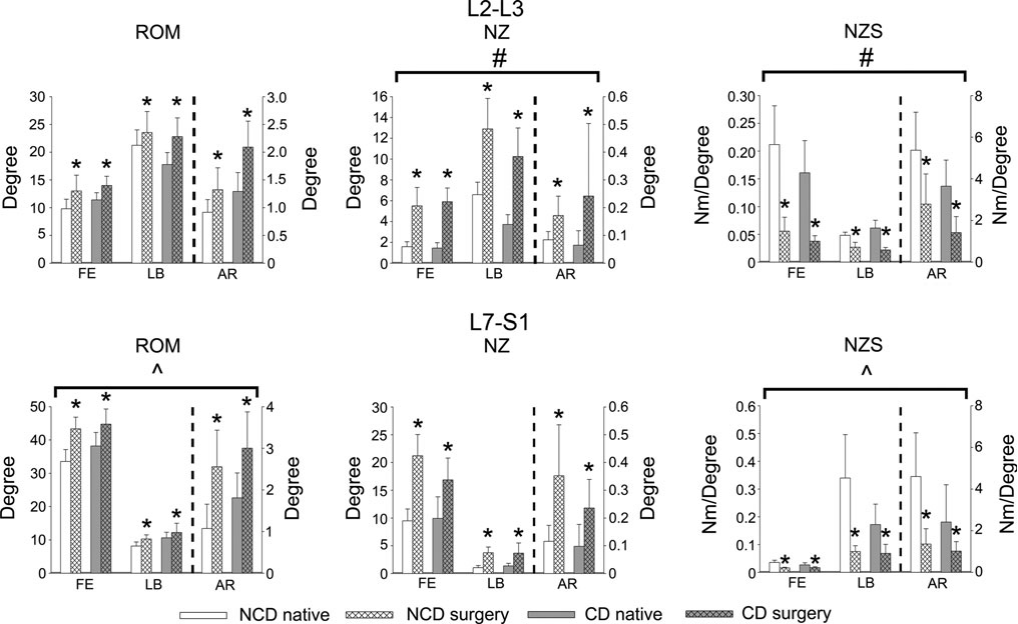
Mean ± SD of the range of motion (ROM), neutral zone (NZ), and neutral zone stiffness (NZS) of the segments L2–L3 (nucleotomy) and L7–S1 (dorsal laminectomy and nucleotomy) in flexion/extension (FE), lateral bending (LB), and axial rotation (AR) for the native state and after surgery in spines from non-chondrodystrophic (NCD) dogs and chondrodystrophic (CD) dogs. The *left* vertical axes apply to FE and LB, and the *right* one to AR (indicated by *dashed line*). * Indicates significant (*P* < 0.05) effect of surgery; ^#^ and ^ indicate significantly greater effect of surgery on CD and NCD spines, respectively

No significant differences were found between the native state and the operated state for the control segments L1–L2 and L6–L7 in both groups (data not shown); however, the ROM, NZ, and NZS were markedly different in the spinal segments L2–L3 and L7–S1, which had undergone nucleotomy (Table 2). Nucleotomy at L2–L3 significantly increased the ROM_FE_, ROM_LB_, and ROM_AR_ to a similar extent in NCD and CD spines (Figs. 3, 4). Nucleotomy significantly increased the NZ_FE_, NZ_LB_, and NZ_AR_, and significantly decreased the NZS_FE_, NZS_LB_, and NZS_AR_, with a significantly larger increase in NZ and significantly larger decrease in NZS in the CD spines than in the NCD spines in all directions of motion.

**Table 2:** Statistical analyses results

	L2–L3			L7–S1		
	ROM	NZ	NZS	ROM	NZ	NZS
Condition	<0.001	<0.001	<0.001	<0.001	<0.001	<0.001
Dog type	<0.001	0.001	0.003	<0.001	<0.001	<0.001
MD	<0.001	<0.001	<0.001	<0.001	<0.001	<0.001
Condition × dog type	0.120	0.017	0.043	0.002	0.447	0.004
Condition × MD	<0.001	0.047	0.106	<0.001	0.038	0.004
Condition × dog type x MD	0.1161	0.087	0.293	0.2954	0.353	0.584
Condition per MD						
FE	<0.001	<0.001	<0.001	<0.001	<0.001	<0.001
LB	<0.001	<0.001	<0.001	<0.001	<0.001	<0.001
AR	<0.001	<0.001	<0.001	<0.001	0.001	<0.001
Condition per dog type						
NCD	–	<0.001	<0.001	<0.001^^^	–	<0.001^^^
CD	–	<0.001^#^	<0.001^#^	<0.001	–	<0.001

*P* values for range of motion (ROM), neutral zone (NZ), and neutral zone stiffness (NZS) for the factors ‘condition’ (native or operated), ‘dog type’ (non-chondrodystrophic (NCD) and chondrodystrophic (CD) dogs), ‘motion direction (MD)’ [flexion/extension (FE), lateral bending (LB), and axial rotation (AR)], and their interactions in L2–L3 and L7–S1. In case of significant interactions, *P* values for condition per MD and condition per dog type were calculated. *P* < 0.05 was considered statistically significant

^#^ Significantly higher effect of the factor condition in CD spines compared with NCD spines

^^^ Significantly higher effect of the factor condition in NCD spines compared with CD spines

Dorsal laminectomy and nucleotomy of L7–S1 significantly increased the ROM_FE_, ROM_LB_, and ROM_AR_, with a significantly larger increase in the NCD spines than in the CD spines in all directions of motion. Surgery resulted in a significant increase in NZ_FE_, NZ_LB_, and NZ_AR_, with no significant differences between the NCD and CD spines. Dorsal laminectomy and nucleotomy significantly decreased the NZS_FE_, NZS_LB_, and NZS_AR_, with a significantly larger decrease in the NCD spines than in the CD spines in all directions of motion.

## Discussion

The biomechanical properties of two types of dog spines, which differed regarding the state of IVD degeneration, were investigated in the native state and after decompressive surgery. The dog offers an interesting model to study the effects of IVD degeneration, with the CD and NCD dogs showing early and late spontaneous degeneration, respectively. The tested specimens were subjected to a cyclic bending moment of −2 to 2 Nm. Although this bending moment is considerably smaller than the moment applied when biomechanically testing human lumbar spinal specimens (±7.5 Nm [15]), this applied moment was assessed to be physiological for the relatively small canine specimens. Using the bending moment of −2 to 2 Nm, the specimens were moved over their complete physiological range of motion without being damaged. In both the NCD and CD spines, the caudal lumbar segments, especially L7–S1, were most mobile in FE, whereas the cranial lumbar segments were more mobile in LB; all segments were relatively stiff in AR, with L7–S1 being the most mobile. It is not possible to directly compare our data for dog spines with those for human spines because of differences in test set-up. However, several similarities and differences can be observed. One notable difference is that normal dog spines have 7 lumbar vertebrae, whereas normal human spines only have 5. The intersegment differences in FE, LB, and AR found in the present study are also seen in the human spine. The absolute mobility of the L1–L2 and L2–L3 segments in FE appears to be comparable in canine and human spines, whereas the canine spine is more mobile in LB. The mobility of the L6–L7 segment in FE and LB in dogs is comparable to that of the L4–L5 segment in humans. Although the mobility of the L7–S1 segment in dogs in LB is similar to that of the L5–S1 segment in humans, it is considerably more mobile in FE; in addition, the canine lumbar spine is considerably stiffer than the human spine in AR [19]. All in all, the canine lumbar spine resembles the human spine biomechanically, but caution is warranted regarding the absolute mobility of the individual spinal segments.

When using the dog as a model for spinal research, both CD and NCD dogs have their specific qualities. CD dogs show degeneration of all IVDs before 2 years of age, allowing biomechanical research into the degenerated IVD and novel strategies for treating the degenerated IVD [5, 8, 9]. In NCD dogs, IVDs remain healthy and rich in notochordal cells until late in life [5, 8–10]. Therefore, NCD dogs allow biomechanical research into the healthy, notochordal cell-rich IVD.

In the present study, the NP of all NCD spines was gelatinous (Thompson grade I), whereas the NP of all CD spines was fibrocartilaginous (Thompson grade II). In the human spine, an increasing grade of IVD degeneration is associated with an increase in segmental ROM, especially in AR [20, 21]. Therefore, comparison of the native spine from CD dogs and NCD dogs could provide novel insights into the effects of IVD degeneration and could be used to assess the validity of the dog as a model of spontaneous IVD degeneration. We initially aimed to make this comparison, using a correction factor for size differences between the NCD and CD dogs based on the transverse area and height of the IVDs in both types of dog. This analysis indicated that the degenerated CD spine exhibited a larger ROM in AR (in line with human studies [20, 21]) and that the NCD, notochordal cell-rich IVD exhibited a larger NZ (data not shown). However, comparison of the NCD spine with the smaller CD spine, using such a correction factor, is problematic because of the non-linearity of the load–displacement behavior of the spines, and differences in the geometry of the facet joints and other spinal structures between the two types of dog. We, therefore, focused on the effects of decompressive surgery on the biomechanical properties of NCD and CD spines, using each spine as an intrinsic control. Decompressive surgery resulted in an increased ROM and NZ, and decreased NZS in all directions of motion in both groups of dogs, as has been reported in humans [12, 22]. However, nucleotomy of L2–L3 resulted in a significantly larger increase in NZ and decrease in NZS in FE, LB, and AR in the CD spines than in the NCD spines, whereas dorsal laminectomy and nucleotomy of L7–S1 resulted in relatively greater increases in the ROM and greater decreases in NZS in FE, LB, and AR in the NCD spines than in the CD spines. These differences indicate that there are substantial biomechanical differences between NCD and CD spines. Apart from differences in the state of IVD degeneration, these differences may be caused by differences in the size/weight of the types of dog, IVD height and size relative to the size of the dog, the facet joint orientation and conformation, and other spinal characteristics [23]. These differences require further investigation.

The present study had some limitations. Although the Beagle dog and mongrel dog spines were of the CD and NCD types, respectively, they represent one group/breed of dogs. Therefore, because several other dog breeds can be classified as NCD or CD, the data obtained cannot be generalized to all NCD and CD dogs.

In conclusion, the biomechanics of the native canine spine differs by spinal level, and in both NCD and CD dogs spinal surgery results in a significant decrease in stiffness similar to that seen in the human spine after decompressive surgery. In view of using the dog as a model for biomechanical research of the spine, NCD dogs can serve as an appropriate model for studying the healthy, notochordal cell-rich IVD, while CD dogs are appropriate for studying degenerated IVDs. Spinal biomechanics and the effects of spinal surgery significantly differ between NCD and CD dogs as a result of existing IVD degeneration in CD dogs. This knowledge should be taken into account when using the dog as a model for spinal research.
